# Acceptable clinical outcomes despite high reoperation rate at minimum 12-month follow-up after concomitant arthroscopically assisted anterior cruciate ligament reconstruction and medial meniscal allograft transplantation

**DOI:** 10.1186/s43019-023-00176-4

**Published:** 2023-01-10

**Authors:** Dhruv S. Shankar, Kinjal D. Vasavada, Amanda Avila, Brittany DeClouette, Hadi Aziz, Eric J. Strauss, Michael J. Alaia, Laith M. Jazrawi, Guillem Gonzalez-Lomas, Kirk A. Campbell

**Affiliations:** Department of Orthopedic Surgery, New York University Langone Health, 333 East 38th St, 4th Floor, New York, NY 10029 USA

**Keywords:** Anterior cruciate ligament reconstruction, Meniscus allograft transplantation, Arthroscopy, Outcomes, PROMIS

## Abstract

**Background:**

Single-stage medial meniscus allograft transplantation (MAT) with concomitant anterior cruciate ligament reconstruction (ACLR) is a technically challenging procedure for management of knee pain and instability in younger patients, but clinical and functional outcomes data are sparse. The purpose of this study was to assess surgical and patient-reported outcomes following concomitant ACLR and medial MAT.

**Methods:**

We conducted a retrospective case series of patients who underwent medial MAT with concomitant primary or revision ACLR at our institution from 2010 to 2021 and had minimum 12-month follow-up. Complications, reoperations, visual analog scale (VAS) pain, satisfaction, Lysholm score, return to sport, and return to work outcomes were assessed. Patient-Reported Outcomes Measurement Information System (PROMIS) Pain Interference, Pain Intensity, and Physical Function Scores were used to measure patients’ functional status relative to the US population. *P*-values < 0.05 were considered significant.

**Results:**

The cohort consisted of 17 knees of 16 individual patients. The cohort was majority male (82.4%) with mean age of 31.9 years (range 19–49 years) and mean body mass index (BMI) of 27.9 kg/m^2^ (range 22.5–53.3 kg/m^2^). Mean follow-up time was 56.8 months (range 13–106 months). Most patients underwent revision ACLR (64.7%). The 1-year reoperation rate was high (23.5%), with two patients (11.8%) tearing their meniscus graft. Patient-reported outcomes indicated low VAS pain (mean 2.2), high satisfaction (mean 77.9%), and fair Lysholm score (mean 81.1). Return to work rate was high (92.9%), while return to sport rate was low (42.9%). Postoperative PROMIS scores were comparable or superior to the national average and correlated significantly with patient satisfaction (*p* < 0.05).

**Conclusions:**

The concomitant ACLR and MAT procedure is associated with excellent knee pain and functional outcomes and high rate of return to work after surgery, though the 1-year reoperation rate is high and rate of return to sport is low.

*Level of evidence*: IV.

## Background

Meniscus allograft transplantation (MAT) is one of several operative treatments available for the management of meniscus deficiency in symptomatic patients younger than 50 years of age. While meniscus allografts are relatively durable with a 10-year survival rate of 73.5% and a 20-year survival rate of 56.2%, almost 32% require reoperation at some point [1, 2]. In those patients presenting with knee instability in the setting of meniscus deficiency, a concomitant anterior cruciate ligament reconstruction (ACLR) may be performed to restore normal knee kinematics and reduce abnormal loading of the transplanted meniscus [3, 4].

ACLR and MAT may sometimes be performed as separate procedures due to the challenge of achieving precise positioning, tensioning, and fixation of both grafts. However, this subjects the patients to two procedures and two separate rehabilitations. While various techniques have been described in the literature for performing ACLR and MAT as a single-stage arthroscopic procedure, the combined procedure is technically demanding [4, 5]. Over the past two decades, a few case series and retrospective cohort studies have been conducted to assess outcomes of concomitant ACLR and MAT, and most have reported substantial improvements in patient-reported outcome measures, as well as high rates of graft survival and return to sport at 3–5 year follow-up [6–10]. Nonetheless, outcomes data for US patients undergoing combined ACLR and MAT remain scarce, especially outcomes collected using survey instruments calibrated against the US population at large, such as the Patient-Reported Outcomes Measurement Information System (PROMIS) [11]. Population-calibrated outcome scores are important for assessing procedure success since they provide a measure of how patients are performing in terms of pain and physical function relative to the average individual.

The aims of our study were to (1) characterize rates of complications and adverse events following concomitant ACLR and medial MAT, (2) assess knee pain, function, and other clinical outcomes following this procedure, (3) assess return to sport and return to work outcomes, and (4) identify associations between PROMIS scores and other patient-reported outcomes in this population.

## Methods

### Study design and ethical approval

We conducted a single-center, multi-surgeon retrospective case series of patients who underwent concomitant arthroscopic ACLR and medial MAT procedures. Institutional review board approval was obtained prior to commencing any study procedures.

### Cohort selection and eligibility criteria

We identified patients who underwent concomitant arthroscopic ACLR and medial MAT procedures by one of six sports medicine fellowship-trained surgeons at our institution from 1 January 2010 to 31 December 2021. We obtained an initial list of patients by searching our electronic medical record system using Current Procedural Terminology (CPT) codes for patients who underwent both primary or revision ACLR (CPT code 29888) and MAT (CPT code 29868). Patients on the list were then individually screened for eligibility. Inclusion criteria were (1) primary or revision ACLR with MAT of the medial meniscus, (2) at least 18 years of age, and (3) minimum 12 months of follow-up. The 12-month cutoff for follow-up was selected since the standard post-ACLR rehabilitation protocol at our institution discourages return to sport prior to 6–8 months postoperation. Exclusion criteria were (1) ACLR and medial MAT performed as separate procedures on different dates, (2) lateral MAT, (3) revision MAT, (4) concomitant realignment procedure such as high tibial osteotomy (HTO), or (5) medial meniscus procedures besides MAT such as meniscus repair or meniscectomy.

At our institution, indications for MAT were (1) age < 50 years, (2) pain with physical activity, (3) well-aligned knee with axial malalignment < 2° of deviation to the involved compartment, (4) primary meniscal deficiency or deficiency secondary to a prior meniscectomy, (5) knee osteoarthritis of Kellgren–Lawrence (KL) grade 2 or less, and (6) intact articular cartilage or focal chondral defects without diffuse chondromalacia. Contraindications to MAT were (1) osteoarthritis of KL grade 3 or 4, (2) osteophyte formation and/or chondral flattening over the tibial condyles that could prevent adequate seating of the graft, (3) skeletal immaturity, (4) synovial disease, (5) inflammatory arthritis, and (6) prior joint infection.

Patients could also undergo a concomitant osteochondral allograft (OCA) procedure to address chondral pathology. Indications for OCA at our institution are: (1) age < 50 years, (2) symptoms of knee pain, swelling, and/or catching, (3) physically active with participation in highly demanding sports/activities, and (4) full-thickness chondral defect of diameter 15–35 mm.

### Surgical technique

All cases used fresh frozen, non-irradiated double bone plug meniscal allografts for transplantation that were size matched for the patient on the basis of magnetic resonance imaging (MRI). Bone plugs were appropriately sized and tubularized on the back table. For the ACLR portion, either bone patellar tendon bone (BPTB) autograft or allograft, hamstring autograft, or tibialis anterior allograft were utilized. For revision ACLR procedures, hamstring autografts were avoided due to their association with tunnel widening compared to other graft types [12].

After graft preparation, standard diagnostic knee arthroscopy was performed. In the lateral compartment, any pathology of the lateral meniscus was addressed via repair and/or debridement at this time. In the medial compartment, the remaining meniscus was debrided to a 2 mm peripheral rim to provide a firm attachment site and prevent extrusion. Attention was first focused on drilling of the femoral tunnel for the ACLR portion. Remnant ACL was debrided followed by preparation of the femoral tunnel by medial portal drilling or retrograde reaming with a standard drill.

The posterior meniscal root insertion was then addressed. A multi-use guide was used to drill the posterior tunnel followed by retrograde reaming with a 9 mm reamer. This was followed by creation of a tibial tunnel for the ACLR, which was reamed with a 10 mm reamer. The position of the tibial tunnel was adjusted accordingly to provide space for the future tunnel drilled for the anterior meniscus bone plug; this was achieved by drilling obliquely or using a lateral entry point distally on the tibia to minimize potential overlap with the bone plug tunnel [4]. Passage of the meniscus allograft was performed using shuttling sutures through a medial safety incision where care was taken to preserve the saphenous vein. The posterior plug was docked and secured with a suture button while FasT-Fix all-inside sutures (Smith & Nephew, Andover, MA, USA) were used to secure the posterior horn. Multiple inside-out suture tapes were passed to secure the body and anterior horn in place.

**Fig. 1:**
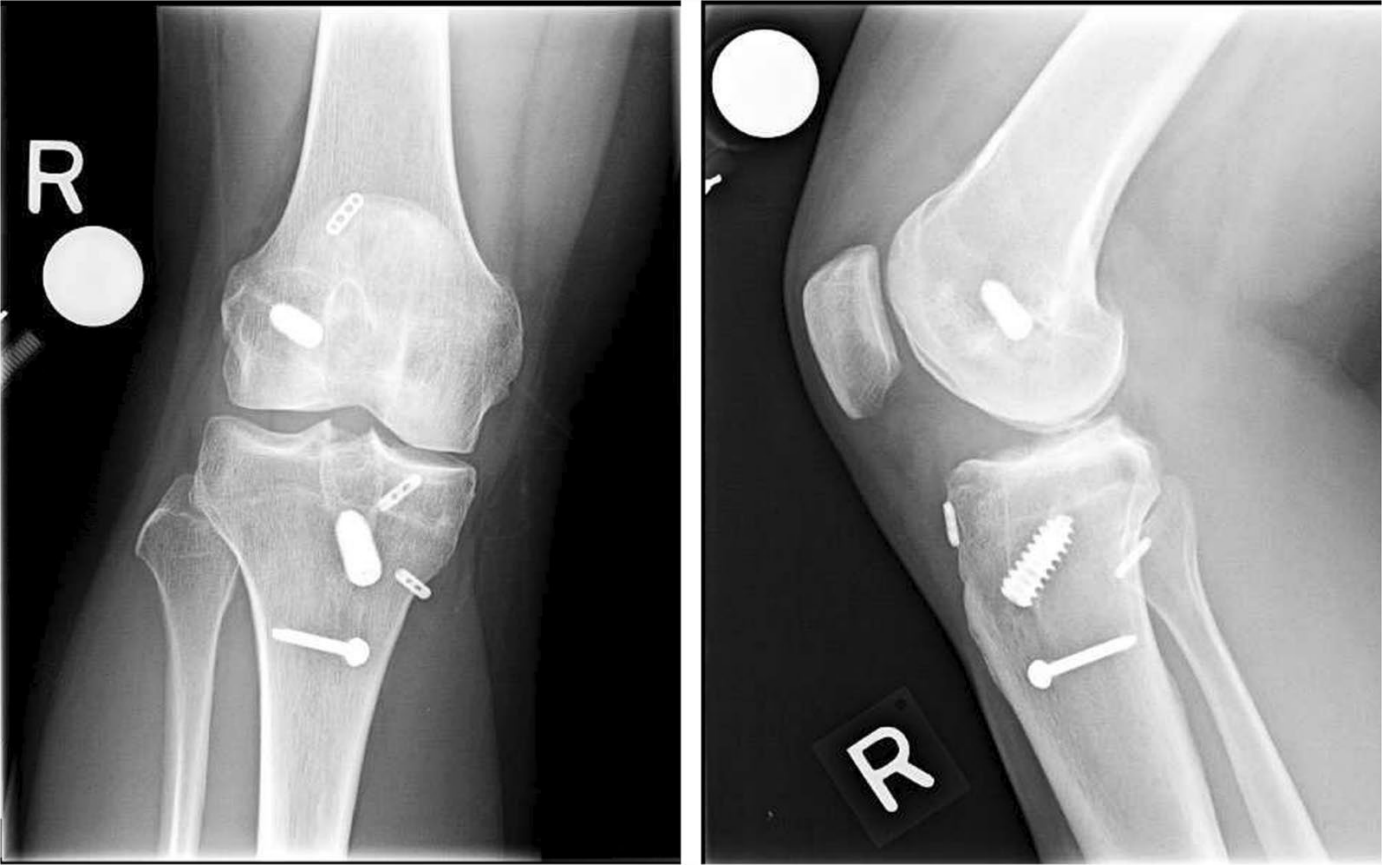
Knee X-rays with AP view (left) and lateral view (right) taken at 17 months following medial MAT and revision ACLR

The ACL graft was passed into the joint in standard fashion. The anterior meniscus root insertion was prepared by creating a tunnel at the attachment site using a 9 mm reamer and carefully examining the tunnel to ensure there was an adequate bone bridge between the attachment site and ACL tibial tunnel. The anterior bone plug was then docked and fixed with a suture button. The knee was cycled to test stability of the ACL and meniscus grafts. Once deemed acceptable, final fixation of the ACL graft was achieved with a metal interference screw. Following the ACLR and MAT procedures, any additional procedures to address cartilage or extra-articular ligamentous pathologies were performed. Postoperative X-rays taken at 17 months following revision ACLR and medial MAT using the described technique are depicted in Fig. 1.

### Postoperative protocol

All patients were instructed to follow a three-phase rehabilitation protocol. During phase I (weeks 1–8), patients were restricted to partial weight bearing in a hinged knee brace locked in extension for weeks 1–2, advanced to weight bearing as tolerated with flexion limited to 90° for weeks 2–6, and discontinuation of the knee brace and progression to full range of motion (ROM) for weeks 6–8. During the first 4 weeks, patients also performed straight-leg raise exercises with the brace in full extension in order to develop quadriceps strength and prevent extensor lag. During phase II (weeks 8–12), patients progressed to closed chain extension, hamstring strengthening, and proprioception exercises. During phase III (months 3–6), patients continued quadriceps and hamstring strengthening exercises with a focus on single-leg strength, began jogging and running, and participated in plyometrics and sport-specific drills. A gradual return to athletic activity was permitted at 6 months postoperation. Second-look diagnostic arthroscopy was not performed unless there was concern for ACL or meniscus graft injury based on clinical and radiographic findings. Postoperative X-rays and MRIs were obtained at the surgeon’s discretion on the basis of the patient’s postoperative course and individual risk factors for graft failure.

### Demographic and operative variables

Patient demographics, surgical history of the index knee, and operative data were abstracted from electronic medical records. Kellgren–Lawrence (KL) osteoarthritis grade of the index knee was obtained from preoperative X-rays. Concomitant procedures besides the ACLR and medial MAT (e.g., osteochondral allograft) were abstracted from intraoperative reports. Complications within 90 days of index surgery were recorded. Any adverse events that occurred between the index surgery and last follow-up were also noted; these events included 90-day complications, tear of the ACL graft, tear of the meniscus allograft, chondral injury, arthrofibrosis, reoperation, and conversion to total knee arthroplasty (TKA).

### Outcomes measured

Postoperative outcomes were assessed using a survey distributed to all eligible subjects in the cohort via email using the REDCap electronic data capture tool [13, 14]. Subjects who underwent bilateral surgery were asked to complete separate surveys for the left and right knee and each knee was treated as a separate subject in the analysis. The primary outcomes were: current knee pain rated on a 10-point visual analog scale (VAS), overall satisfaction with the procedure on a 100-point scale, current knee function rated using the Lysholm Knee Scoring Scale, and highest level of physical activity prior to injury, prior to surgery, and at present using the Tegner Activity Scale. The “locking/catching sensation” question and “instability” question from the Lysholm survey were used to assess rates of postoperative knee locking/catching and knee instability.

Secondary outcomes included return to sport (RTS) and return to work (RTW). For RTS, patients reported whether they participated in sports prior to injury, whether they were able to return to sports after surgery, and whether they returned to their pre-injury level of competition or higher. For RTW, patients reported whether they were employed prior to injury, the physical intensity of their work according to the Association for Work Design, Business Organization and Business Development (REFA) classification of workload (Table 1) [15], and whether they were able to return to work after surgery.

**Table 1:** Association for Work Design, Business Organization and Business Development (REFA) classification of work intensity

Grade	Definition
0	Working without carrying loads (e.g., desk work, office work)
1	Handling light work pieces, walking or standing around for long periods
2	Carrying loads of 20–30 lbs (10–15 kg), climbing stairs or ladders without load
3	Carrying loads of 40–60 lbs (20–30 kg), climbing stairs or ladders with moderate load, working in a tense posture
4	Carrying loads of more than 110 lbs (50 kg), climbing with heavy loads, hard work

Patients also completed the Patient-Reported Outcomes Measurement Information System (PROMIS) Pain Interference v1.1, Pain Intensity Short Form 3a v1.0, and Physical Function v2.0 computerized adaptive tests (CATs). PROMIS instruments are scored using the general US population as a reference distribution; the mean population score is set at 50 and the population standard deviation is set at 10 [16]. Higher scores indicate that the patient is experiencing more of the attribute measured by the instrument (e.g., higher Pain Intensity score means more intense pain). PROMIS CATs are administered preoperatively and at follow-up visits as part of the standard of care at our institution. Therefore, for patients who did not complete the email survey, postoperative PROMIS scores were instead abstracted from the latest follow-up visit note.

### Statistical analysis

All analyses were conducted on each individual knee and subjects undergoing bilateral procedures were treated as two separate subjects. Descriptive statistics were calculated for demographics, operative variables, postoperative events, and clinical outcomes. Continuous variables were assessed for normality using the Shapiro–Wilk test and found to be non-normally distributed. Changes in Tegner score between the pre-injury baseline and present day and the preoperative baseline and present day were evaluated using the Wilcoxon signed rank test. A subgroup analysis was performed to compare demographics, operative characteristics, and outcomes between patients who underwent MAT with primary ACLR versus revision ACLR using the Mann–Whitney *U* test for continuous variables and Fisher’s exact test for categorical variables. Bivariate associations between PROMIS scores and the VAS pain, satisfaction, and Lysholm scores were assessed using the Spearman rank correlation coefficient (*⍴*), and the significance of these associations was determined using hypothesis testing with the *t*-statistic. *P*-values < 0.05 were considered significant.

## Results

### Cohort demographics

**Fig. 2:**
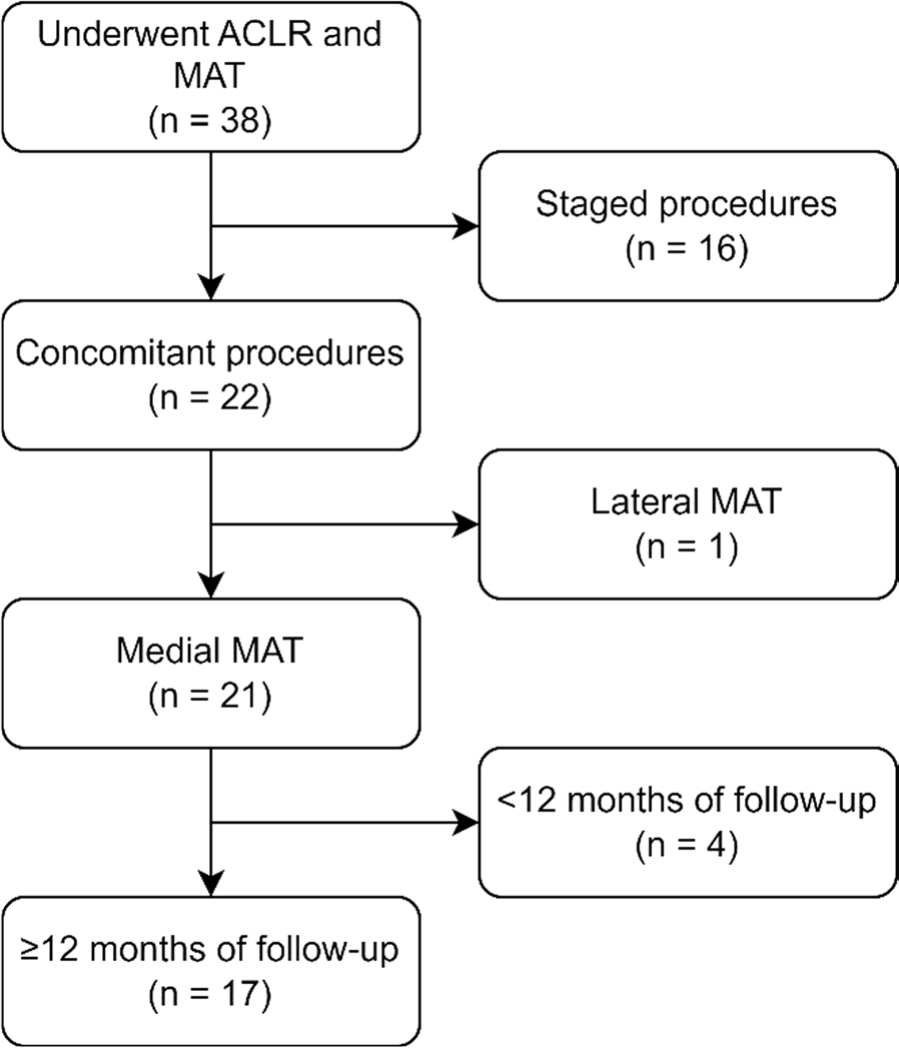
Patient flow through the study

Patient flow is summarized in Fig. 2. On initial search, 38 patients were identified as having undergone ACLR and MAT procedures. Of these, 16 patients were excluded for not having concomitant ACLR and MAT procedures, 1 patient was excluded for having lateral MAT, and 4 patients were excluded for having less than 12 months of follow-up. The final cohort consisted of 17 knees of 16 individual patients (15 unilateral procedures, 1 bilateral procedure).

Cohort demographics are presented in Table 2. The cohort was majority male (15 patients, 82.4%) with mean age of 31.9 years (range 19–49 years) and mean body mass index (BMI) of 27.9 kg/m^2^ (range 22.5–53.3 kg/m^2^). Seven patients (41.2%) had no radiographic evidence of knee osteoarthritis while the remainder were KL grade 1 (11.8%) or grade 2 (47.1%). A majority of the cohort (13 patients, 76.5%) had prior non-ACLR surgery on the index knee, most commonly medial arthroscopic partial meniscectomy (APM) (13 patients, 76.5%). Among patients who did not undergo medial APM, the indication for meniscus transplant was primary meniscus deficiency due to tear or chronic degeneration in addition to the ACL deficiency. The mean follow-up time was 56.8 months (range 13–106 months).

**Table 2:** Demographic information

Variable	All (*n* = 17)	Primary ACLR (*n* = 6)	Revision ACLR (*n* = 11)	*P*-value
Age^†^	31.9 ± 7.6	34.3 ± 9.2	30.6 ± 6.7	0.36
Sex				0.51
Male	14 (82.4%)	6 (100.0%)	8 (72.7%)	
Female	3 (17.7%)	0 (0.0%)	3 (27.3%)	
BMI^†^ (kg/m^2^)	27.9 ± 7.2	30.5 ± 11.5	26.5 ± 3.3	1.00
Race				0.003*
White or Caucasian	11 (64.7%)	1 (16.7%)	10 (90.9%)	
Black or African-American	2 (11.8%)	1 (16.7%)	1 (9.1%)	
Asian and/or Pacific Islander	3 (17.7%)	3 (50.0%)	0 (0.0%)	
Other	1 (5.9%)	1 (16.7%)	0 (0.0%)	
Smoking history				1.00
Never smoker	10 (58.8%)	3 (50.0%)	7 (63.6%)	
Former smoker	2 (11.8%)	1 (16.7%)	1 (9.1%)	
Current smoker	5 (29.4%)	2 (33.3%)	3 (27.3%)	
ASA physical status classification				0.55
1	10 (58.8%)	3 (50.0%)	7 (63.6%)	
2	6 (35.3%)	2 (33.3%)	4 (36.4%)	
3	1 (5.8%)	1 (16.7%)	0 (0.0%)	
Laterality of index knee				0.64
Left	7 (41.2%)	3 (50.0%)	4 (36.4%)	
Right	10 (58.8%)	3 (50.0%)	7 (63.6%)	
KL grade of knee osteoarthritis				0.81
0	7 (41.2%)	3 (50.0%)	4 (36.4%)	
1	2 (11.8%)	0 (0.0%)	2 (18.2%)	
2	8 (47.1%)	3 (50.0%)	5 (45.5%)	
Prior non-ACLR index knee surgery	13 (76.5%)	4 (66.7%)	9 (81.8%)	0.58
Medial APM	13 (76.5%)	4 (66.7%)	9 (81.8%)	0.58
Lateral APM	4 (23.5%)	2 (33.3%)	2 (18.2%)	0.58
ACL debridement	4 (23.5%)	2 (33.3%)	2 (18.2%)	0.58
Shaving chondroplasty	4 (23.5%)	2 (33.3%)	2 (18.2%)	0.58
Follow-up time (months)^†^	56.8 ± 31.1	44.2 ± 26.6	63.7 ± 32.4	0.29

*BMI* body mass index, *ASA* American Society of Anesthesiologists, *KL* Kellgren–Lawrence, *APM* arthroscopic partial meniscectomy, *ACL* anterior cruciate ligament

^*^P-value < 0.05

^†^Means reported with standard deviation

Operative characteristics of the concomitant ACLR and medial MAT procedures are presented in Table 3. The majority of patients underwent revision ACLR (11 patients, 66.7%). The most common ACL graft type used was bone–patellar–tendon–bone (BPTB) allograft (ten patients, 58.8%). The most common concomitant procedures were arthroscopic shaving chondroplasty (four patients, 23.5%) and/or removal of hardware (four patients, 23.5%). Following the initial surgery, no patients underwent second-look diagnostic arthroscopy while nine patients (52.9%) underwent MRI imaging of the index knee at least once following their initial surgery. Follow-up time did not significantly differ between the primary and revision ACLR groups (primary 44.2 months versus revision 63.7 months, *p* = 0.29). While there was higher percentage of white patients in the revision ACLR group (primary 16.7% versus revision 90.9%, *p* = 0.003), there were no other statistically significant differences in demographic or operative characteristics between the two subgroups (*p* > 0.05).

**Table 3:** Operative characteristics

Variable	All (*n* = 17)	Primary ACLR (*n* = 6)	Revision ACLR (*n* = 11)	*P*-value
ACL graft type				0.44
BPTB allograft	10 (58.8%)	4 (66.7%)	6 (54.6%)	
BPTB autograft	5 (29.4%)	1 (16.7%)	4 (36.4%)	
Hamstring autograft	1 (5.8%)	1 (16.7%)	0 (0.0%)	
Tibialis anterior allograft	1 (5.8%)	0 (0.0%)	1 (9.1%)	
MAT laterality				1.00
Medial	17 (94%)	6 (100%)	11 (92%)	
Lateral	1 (6%)	0 (0%)	1 (8%)	
Concomitant procedures	12 (70.6%)	4 (66.7%)	8 (72.7%)	1.00
OCA	3 (17.7%)	2 (33.3%)	1 (9.1%)	0.51
Chondroplasty	4 (23.5%)	1 (16.7%)	3 (27.3%)	1.00
Lateral meniscus repair	2 (11.8%)	1 (16.7%)	1 (9.1%)	1.00
Lateral APM	1 (5.9%)	0 (0.0%)	1 (9.1%)	1.00
Removal of hardware	4 (23.5%)	1 (16.7%)	3 (27.3%)	1.00
ALL reconstruction	1 (5.9%)	0 (0.0%)	1 (9.1%)	1.00

*ACLR* anterior cruciate ligament reconstruction, *ACL* anterior cruciate ligament, *BPTB* bone–patellar–tendon–bone, *MAT* meniscus allograft transplantation, *APM* arthroscopic partial meniscectomy, *OCA* osteochondral allograft, *ALL* anterolateral ligament

### Postoperative complications and adverse events

Two patients (11.8%) experienced a complication within 90 days of surgery. The first patient was a 44-year-old male who underwent MAT with revision ACLR who first complained of numbness over the medial pretibial area at 2 weeks postoperation. This progressed to constant burning pain over the anterior and anteromedial pretibial area and medial calf by 4 weeks postoperation. On the basis of the distribution of the paresthesia, the patient was diagnosed with saphenous neuropathy, possibly secondary to nerve injury from the medial incision site. The same patient also developed arthrofibrosis with knee flexion limited to 90° by 9 weeks postoperation necessitating manipulation under anesthesia (MUA) at 3 months postoperation. At last follow-up, he no longer complained of stiffness and was able to flex the knee to 120° but was still experiencing neuropathic pain. The second patient was a 37-year-old male who underwent MAT with primary ACLR, developed arthrofibrosis with knee flexion limited to 100° by 7 weeks postoperatively, and underwent MUA at 3 months postoperation. At last follow-up, he no longer complained of stiffness and was able to flex the knee to 130°.

Nine patients (52.9%) experienced at least one adverse event by last follow-up with four patients (23.5%) experiencing these events within 1 year of index surgery. Two patients (11.8%), one primary and one revision ACLR, experienced a tear of their medial meniscus allograft. The revision ACLR patient was asymptomatic but was found to have a posterior root tear on a screening MRI taken at 5 months postoperative and underwent arthroscopic repair 1 week later. The primary ACLR patient began experiencing recurrence of pain in the index knee at 10 months postoperative, was referred for an MRI, which revealed a posterior horn tear, and was recommended to undergo arthroscopic repair but ultimately declined further surgery. At last follow-up, no patients required surgical removal of their meniscus graft.

Two patients (11.8%), both revision ACLRs, experienced a tear of their ACL graft at 8 months and 21 months postoperation and both underwent another revision ACLR. Three patients (17.6%) developed arthrofibrosis; two were discussed previously, while the third as diagnosed at 23 months postoperation but declined further intervention. One patient (5.9%), a 34-year-old male who underwent revision ACLR, was asymptomatic at 26 months postoperation when he experienced trauma to the index knee resulting in new onset pain and was found to have a complex lateral meniscus tear and a full-thickness lateral femoral condyle (LFC) chondral defect on MRI. He underwent lateral APM and LFC chondroplasty at 41 months postoperation. One patient (5.9%), a 26-year-old male with no prior radiographic evidence of knee osteoarthritis (KL grade 0) who underwent revision ACLR, developed post-traumatic osteoarthritis following index surgery associated with progressive localized medial and lateral knee pain and was found to have KL grade 2 osteoarthritic changes on X-ray at 35 months postoperation. He underwent TKA at 47 months postoperation.

In total, seven patients (41.1%) underwent further operative interventions for sequelae related to the index procedure at an average of 18.1 months postoperation (range 2–47 months) with four reoperations (23.5%) occurring within 1 year of index surgery. As described previously, reoperations included two MUAs, two revision ACLRs, one lateral APM and LFC chondroplasty, one medial meniscus allograft root repair, and one TKA. Reoperations were more common among revision ACLR patients (5 of 11, 45.5%) compared with primary ACLR patients (1 of 6, 16.7%), but given the available sample size, this difference was not found to be statistically significant (*p* = 0.33).

### Clinical outcomes, return to sport, and return to work

Clinical outcomes at the latest follow-up visit are presented in Table 4. There were 14 responses to the email survey. On average, VAS knee pain levels were low (mean 2.2) and procedure satisfaction scores were high (mean 77.9%). Mean Lysholm score was 81.1 which corresponds to a “fair” rating [17]. Rate of knee catching was low (2 patients, 14.3%), and no patients complained of locking symptoms. Rate of knee instability during sports, as indicated by the Lysholm survey, was high (11 patients, 78.6%). Only one patient complained of instability during activities of daily living (7.1%), though there was no clinical evidence of knee instability noted on their last postoperative physical examination. While Tegner scores improved significantly improved between the post-injury period and present (mean post-injury 3.9 to present 4.9, *p* = 0.005), but present-day scores were still significantly lower than the pre-injury baseline (mean present 4.9 to pre-injury 7.4, *p* = 0.04).

**Table 4:** Patient-reported outcomes and return to work and sport

Survey responses	All (*n* = 14)	Primary ACLR (*n* = 4)	Revision ACLR (*n* = 10)	*P*-value
VAS pain^†^	2.2 ± 3.3	2.8 ± 4.8	1.9 ± 2.8	0.89
Satisfaction^†^	77.9% ± 35.5%	100.0% ± 0.0%	69.0% ± 38.9%	0.04*
Lysholm score^†^	81.1 ± 18.1	86.5 ± 14.5	78.9 ± 19.7	0.52
Knee catching	2 (14.3%)	0 (0.0%)	2 (20.0%)	1.00
Knee locking	0 (0.0%)	0 (0.0%)	0 (0.0%)	1.00
Knee instability during sports	11 (78.6%)	3 (75.0%)	8 (80.0%)	1.00
Knee instability during ADLs	1 (7.1%)	0 (0.0%)	1 (10.0%)	1.00
Tegner scores^†^				
Pre-injury	7.4 ± 2.5	7.0 ± 3.6	7.5 ± 2.2	1.00
Post-injury	3.9 ± 2.4	4.8 ± 1.5	3.5 ± 2.6	0.32
At present	4.9 ± 2.1	5.0 ± 1.4	4.8 ± 2.4	0.88
Change from pre-injury	−2.5 ± 2.7	−2.0 ± 2.3	−2.7 ± 2.9	0.62
Change from post-injury	1.0 ± 1.7	0.3 ± 0.5	1.3 ± 1.9	0.25
Participated in sports prior to injury	14 (100.0%)	4 (100.0%)	10 (100.0%)	1.00
Full- or limited-contact sport	4 (28.6%)	0 (0.0%)	4 (40.0%)	0.25
Returned to sport after surgery	6 (42.9%)	2 (50.0%)	4 (40.0%)	1.00
Returned at the pre-injury level	6 (42.9%)	2 (50.0%)	4 (40.0%)	1.00
Employed prior to injury	14 (100.0%)	4 (100.0%)	10 (100.0%)	1.00
REFA work intensity class				0.84
0	1 (7.1%)	0 (0.0%)	1 (10.0%)	
1	5 (35.7%)	1 (25.0%)	4 (40.0%)	
2	0 (0.0%)	0 (0.0%)	0 (0.0%)	
3	4 (28.6%)	2 (50.0%)	2 (20.0%)	
4	4 (28.6%)	1 (25.0%)	3 (30.0%)	
Returned to work after surgery	13 (92.9%)	4 (100.0%)	9 (90.0%)	1.00
Returned at the pre-injury level	9 (64.3%)	2 (50.0%)	7 (70.0%)	0.53

Postoperative PROMIS CAT scores	All (*n* = 16)	Primary ACLR (*n* = 5)	Revision ACLR (*n* = 11)	*P*-value
Pain interference^†^	51.8 ± 11.5	49.7 ± 10.5	52.8 ± 12.2	0.82
Pain intensity^†^	40.8 ± 9.2	38.9 ± 8.9	41.6 ± 9.6	0.73
Physical function^†^	53.1 ± 11.8	60.3 ± 12.3	50.2 ± 10.9	0.23

*VAS* visual analog scale, *ADLs* activities of daily living, *REFA* Association for Work Design, Business Organization and Business Development, *PROMIS* Patient-Reported Outcomes Measurement Information System, *CAT* computerized adaptive test

^*^P-value < 0.05

^†^Means reported with standard deviation

All survey respondents (*n* = 14) participated in at least one sport or physical activity prior to injury. Four subjects (28.6%) participated in full- or limited-contact sports such as field hockey, Brazilian jiu-jitsu, soccer, and basketball. Of the 14 respondents, 6 (42.9%) returned to sport after surgery and all 6 returned to sport at or above their pre-injury level of competition. The eight subjects who did not return to sport cited the following reasons: recovery from the surgery (five of eight), lack of confidence (two of eight), and persistent symptoms since the time of injury that were not resolved by surgery (one of eight).

All survey respondents (*n* = 14) were employed prior to injury. A majority were employed in jobs with REFA work intensity grades of 3 or 4 (eight subjects, 57.1%). Of the 14 subjects, 13 (92.9%) returned to work after surgery and 9 (64.3%) returned to work at or above their pre-injury level of intensity. The five subjects who did not return to work or did not return to work at their pre-injury level of intensity cited the following reasons: recovery from the surgery (four of five) and lack of confidence (one of five). Furthermore, all five patients were previously employed in physically strenuous jobs (REFA classification 3 or 4).

Postoperative PROMIS scores were obtained for 16 patients. On average, Postoperative Pain Interference scores (mean 51.8) and Physical Function scores (mean 53.1) were close to the US population mean (50), whereas Pain Intensity scores (mean 40.8) were lower (better) than the US population mean.

Satisfaction scores were significantly lower among revision ACLR patients compared with primary ACLR patients (mean revision 69.0% versus primary 100.0%, *p* = 0.04), but there were no other significant differences in outcomes between the two groups (*p* > 0.05).

### Associations between PROMIS scores and patient-reported outcomes

The Spearman correlation matrix for PROMIS scores versus patient-reported outcomes is displayed in Table 5. Satisfaction score was significantly correlated with all three PROMIS scores: negatively with Pain Interference (*p* = 0.01), negatively with Pain Intensity (*p* = 0.009), and positively with Physical Function (*p* = 0.009). VAS pain was positively correlated with Pain Interference (*p* = 0.04) but had no other significant correlations with PROMIS measures. Lysholm score was not significantly correlated with any of the PROMIS scores (all *p* > 0.05).

**Table 5:** Spearman rank correlations (rho) between PROMIS scores and patient-reported outcomes

	Pain interference	Pain intensity	Physical function
VAS pain	rho = 0.56 *p* = 0.04*	rho = 0.47 *p* = 0.09	rho = −0.47 *p* = 0.09
Satisfaction	rho = −0.66 *p* = 0.01*	rho = −0.67 *p* = 0.009*	rho = 0.67 *p* = 0.009*
Lysholm	rho = −0.27 *p* = 0.35	rho = −0.35 *p* = 0.23	rho = 0.32 *p* = 0.27

*PROMIS* Patient-Reported Outcomes Measurement Information System, *VAS* visual analog scale

^*^P-value < 0.05

## Discussion

### Summary of results

In a cohort of patients who underwent concomitant ACLR and MAT, we observed low levels of patient-reported knee pain and dysfunction, high satisfaction (mean 77.9% out of 100%), and high rate of return to work (92.9%) at mean 4.7-year follow-up. However, there was a high 90-day complication rate (11.8%) and 1-year reoperation rate (23.5%) with over half the cohort (52.9%) experiencing at least one adverse postoperative event by last follow-up. Furthermore, the rate of return to sport was low (42.9%) and a majority endorsed knee instability symptoms during sports and strenuous activity (78.6%) as reported on the Lysholm survey. Only one patient, who underwent medial MAT with revision ACLR, underwent an anterolateral ligament (ALL) reconstruction, and no patient underwent lateral extra-articular tenodesis (LET) for treatment of instability. Furthermore, satisfaction was significantly lower among patients who underwent revision ACLR (mean 69.0%) compared with those who underwent primary ACLR (mean 100%). On the basis of PROMIS scores, patient-reported pain and physical function after surgery among the cohort were comparable to the US population average. Furthermore, PROMIS Pain Interference, Pain Intensity, and Physical Function scores were all significantly correlated with satisfaction scores.

### Knee pain and function

Patients in our cohort exhibited satisfactory outcomes with regards to knee pain and function up to nine years after the concomitant procedure. Several retrospective studies have also reported mid-term and long-term improvements in patient-reported outcomes of knee function following the concomitant ACLR and MAT procedure. Zaffagnini et al. [18] reported significant pre-to-postoperative improvement in VAS and Lysholm scores at mean 5-year follow-up among a cohort of 50 patients who underwent arthroscopic MAT and concomitant ACLR with or without high tibial osteotomy. Sekiya et al. [6] reported normal International Knee Documentation Committee (IKDC) scores at mean 2.8-year follow-up among a cohort of 28 patients who underwent concomitant ACLR and MAT. Yoldas et al. [7] reported mean Lysholm scores of 89.4 ± 8.9 (“good”) among patients undergoing medial MAT with primary ACLR and 74.0 ± 19.1 (“fair”) among undergoing medial MAT with revision ACLR at mean 2.9-year follow-up in a cohort of 31 patients.

### Sports and work outcomes

Our analysis also noted a 42.9% rate of return to sport following concomitant ACL and MAT, with the Lysholm questionnaire revealing that 79% of respondents experienced some degree of knee instability with sports or heavy physical activity. Our cohort exhibited a worse rate of return to sport compared with the Zaffagnini et al. cohort (31 of 38 patients, 81.6%) and a similar rate to a 40-person cohort published by Saltzman et al. with mean 5.7-year follow-up [18, 19]. Furthermore, while all subjects in our cohort who did return to sport did so at or above their pre-injury level, only 48% of subjects in the Zaffagnini et al. cohort and 39% of subjects in the Saltzman et al. cohort were able to do so [18, 19]. The most cited reason among our cohort for failure to return to sport was ongoing recovery from surgery (five of eight, 62.5%) whereas subjects in the Saltzman et al. [19] cohort cited fear of reinjuries (37%) and pain (37%) as their major limitations. With regard to instability symptoms, only one patient in our cohort complained of instability with ADLs and those experiencing instability with sports and strenuous activities did not rate their symptoms as being severe enough to preclude participation in those activities. Likewise, Yoldas et al. [7] found no complaints of instability among patients who underwent MAT with primary ACLR and noted that 63% of patients who underwent MAT with revision ACLR had no or rare instability symptoms with sports. Our cohort also exhibited a similar pattern of Tegner scores to the Zaffagnini et al. [18] cohort in that scores improved significantly between the preoperative and postoperative periods but did not return to the pre-injury baseline, suggesting that, despite the postoperative improvement in functional knee outcomes following ACLR with medial MAT, it may still be insufficient for full return to sport.

Return to work outcomes were excellent among our cohort, with 92.9% of subjects returning to work and 64.3% returning to work at the same or higher level of physical intensity. However, the five patients in our cohort who did not return to work or did not return to work at their prior level of physical intensity were initially employed in jobs involving heavy physical strain as defined by the REFA classification of workload. As with return to sport, the most common limitation cited was recovery from the surgery (four of five, 80.0%). By contrast, the six survey respondents previously employed in jobs involving no or limited physical strain (REFA classes 0 and 1) were all able to return to work at their pre-injury level of intensity. Consequently, returning to work at the pre-injury level after concomitant ACLR and medial MAT may be more feasible for patients originally employed in positions involving a less strenuous physical workload. To our knowledge, no prior studies on outcomes of concomitant ACLR and MAT have reported on pre-injury work intensity or return to work rates among their cohorts. However, Saltzman et al. [19] noted that subjects who experienced meniscus graft failure were more likely to have undergone their procedure as part of a worker’s compensation claim instead of for a sports-related injury. This suggests that there may be differences in mechanisms of injury encountered in certain fields of employment compared with sports, which may impact outcomes after concomitant ACLR and MAT.

### Reoperations and failure rates

While reoperations within 1 year of surgery were common among our cohort (23.5%) and the meniscus graft tear rate was high (11.8%), no patients required removal of the graft by last follow-up and only one patient (5.9%) converted to TKA by final follow-up. These results are consistent with other published studies of concomitant ACLR and MAT outcomes. Yoldas et al. and Sekiya et al. reported no retears or graft failures in their respective cohorts at final follow-up [6, 7]. Zaffagnini et al. [18] reported a 17% reoperation rate and three graft failures (7%) that were all secondary to trauma. Saltzman et al. [19] reported a relatively high failure of 20% with 15% of patients progressing to TKA and 6% of patients requiring revision procedures with full or partial removal of the meniscus graft. On the basis of a subgroup analysis, they found that failed grafts were associated with older age, higher BMI, higher preoperative KL grade, higher baseline patient-reported outcome scores, and not identifying as an athlete. By contrast, the sole patient in our cohort who progressed to TKA had normal BMI (24.4), had no radiographic knee osteoarthritis (KL grade 0) prior to initial surgery, and did identify as an athlete who participated in cycling and hiking. However, this discrepancy should be interpreted in the context of our smaller cohort size compared with the Saltzman et al. cohort.

### PROMIS scores

Our study utilized PROMIS instruments, standardized assessments developed by the National Institutes of Health (NIH) for measuring patient-reported health outcomes across several domains [11]. Unlike traditional outcome measures such as the Lysholm scale, PROMIS instruments are calibrated to reflect the distribution of survey responses observed in the US population at large [11]. Thus, PROMIS scores can be interpreted using a national mean (“the average American”) as a reference. Our cohort exhibited postoperative pain and physical function that was comparable or superior to the national average. Furthermore, we found that patient satisfaction correlated strongly with the PROMIS Pain Interference, Pain Intensity, and Physical Function scores. This finding suggests that these three PROMIS instruments may each serve as a proxy measure for patient satisfaction with their procedure. It should be noted that none of the three PROMIS scores correlated significantly with Lysholm score, which suggests that these instruments may not adequately capture knee-specific outcomes among patients who undergo concomitant ACLR and MAT. While no other studies on concomitant ACLR and MAT outcomes have used PROMIS scores, several have made use of the 36-Item Short Form Survey (SF-36), a physical and mental health outcomes instrument originally developed by the RAND Corporation and that was later normalized against the US population using *z*-scores [20]. Similar to our findings, Yoldas et al. [7] reported that their cohort of ACLR/MAT patients exhibited physical and mental health outcomes comparable or superior to an age- and sex-matched US population, though they were unable to obtain preoperative scores. Likewise, Sekiya et al. [6] found that their cohort was comparable to or outperformed their age- and sex-matched population in most domains of the SF-36 after surgery.

### Primary versus revision ACLR subgroup analysis

While we did not identify significant differences in most pain and functional outcomes between patients undergoing MAT with primary versus revision ACLR, we did find significantly lower satisfaction scores among the latter group. While much of the literature on primary versus revision ACLR has reported significantly worse clinical and patient-reported outcomes among the latter group [21, 22], studies of ACLR/MAT patients have been more inconclusive in this regard. Subgroup analyses by Yoldas et al. and Zaffagnini et al. found no significant differences in postoperative outcomes between primary and revision ACLR groups in their respective cohorts [7, 18]. In comparison, Sekiya et al. [6] noted that primary ACLR patients had significantly better postoperative IDKC scores compared with their revision counterparts in their cohort.

### Limitations

We acknowledge several limitations of our study design. First, the retrospective nature of the study design meant that outcome scores could not be collected preoperatively and that our cohort is subject to selection bias. Second, our cohort included patients from six different surgeons at our center. Though all of them use a similar surgical technique (see section “Surgical technique” under Methods), there may have been variations in graft positioning and other operative details that our analysis could not account for. Third, our subgroup analysis of primary versus revision ACLR patients is limited in statistical power due to the small sample sizes of the groups being compared. It is possible that, with a larger sample size for both groups, additional differences in outcomes could be detected.

## Conclusions

The concomitant ACLR and MAT procedure is associated with excellent knee pain and functional outcomes and high rate of return to work after surgery though the 1-year reoperation rate is high and rate of return to sport is low.

## Data Availability

The datasets used and/or analyzed during the current study are available from the corresponding author on reasonable request.

## Abbreviations

AP: Anterior–posterior
ACL: Anterior cruciate ligament
ACLR: Anterior cruciate ligament reconstruction
ALL: Anterolateral ligament
APM: Arthroscopic partial meniscectomy
ASA: American Society of Anesthesiologists
BMI: Body mass index
BPTB: Bone patellar tendon bone
CPT: Current procedural terminology
IKDC: International Knee Documentation Committee
KL: Kellgren–Lawrence
LET: Lateral extra-articular tenodesis
LFC: Lateral femoral condyle
MAT: Meniscus allograft transplantation
MRI: Magnetic resonance imaging
MUA: Manipulation under anesthesia
OCA: Osteochondral allograft
PROMIS: Patient-Reported Outcomes Measurement Information System
REFA: Association for Work Design, Business Organization and Business Development
RTS: Return to sport
RTW: Return to work
SF-36: 36-Item Short Form Survey
TKA: Total knee arthroplasty
VAS: Visual analog scale
